# A Krill Oil Supplemented Diet Suppresses Hepatic Steatosis in High-Fat Fed Rats

**DOI:** 10.1371/journal.pone.0038797

**Published:** 2012-06-07

**Authors:** Alessandra Ferramosca, Annalea Conte, Lena Burri, Kjetil Berge, Francesco De Nuccio, Anna Maria Giudetti, Vincenzo Zara

**Affiliations:** 1 Department of Biological and Environmental Sciences and Technologies, University of Salento, Lecce, Italy; 2 Aker BioMarine ASA, Oslo, Norway; State University of Rio de Janeiro, Biomedical Center, Institute of Biology, Brazil

## Abstract

Krill oil (KO) is a dietary source of n-3 polyunsaturated fatty acids, mainly represented by eicosapentaenoic acid and docosahexaenoic acid bound to phospholipids. The supplementation of a high-fat diet with 2.5% KO efficiently prevented triglyceride and cholesterol accumulation in liver of treated rats. This effect was accompanied by a parallel reduction of the plasma levels of triglycerides and glucose and by the prevention of a plasma insulin increase. The investigation of the molecular mechanisms of KO action in high-fat fed animals revealed a strong decrease in the activities of the mitochondrial citrate carrier and of the cytosolic acetyl-CoA carboxylase and fatty acid synthetase, which are both involved in hepatic *de novo* lipogenesis. In these animals a significant increase in the activity of carnitine palmitoyl-transferase I and in the levels of carnitine was also observed, suggesting a concomitant stimulation of hepatic fatty acid oxidation. The KO supplemented animals also retained an efficient mitochondrial oxidative phosphorylation, most probably as a consequence of a KO-induced arrest of the uncoupling effects of a high-fat diet. Lastly, the KO supplementation prevented an increase in body weight, as well as oxidative damage of lipids and proteins, which is often found in high-fat fed animals.

## Introduction

Dietary polyunsaturated fatty acids (PUFAs) of the n-3 and n-6 series are potent modulators of the *de novo* fatty acid synthesis in liver [1], [2]. Indeed, PUFAs are able to reduce both the expression and the activity of key enzymes involved in this anabolic pathway, such as the cytosolic acetyl-CoA carboxylase (ACC) and fatty acid synthetase (FAS), thereby leading to a net decrease in the level of newly synthesized fatty acids inside hepatocytes. Furthermore, a diet supplemented with n-3 and/or n-6 PUFAs is able to beneficially influence other aspects of lipid metabolism, such as the levels of circulating triglycerides and cholesterol [3]. Fish oil (FO), a dietary oil enriched in two long chain n-3 PUFAs, eicosapentaenoic acid (EPA, 20:5) and docosahexaenoic acid (DHA, 22:6), is indeed used for its preventive and protective role against cardiovascular diseases [4], [5]. However, in recent years the use of alternative dietary sources of n-3 PUFAs is rapidly spreading amongst population.

Krill oil (KO), a novel dietary supplement extracted from Antarctic krill (*Euphausia superba*), is also rich in EPA and DHA [6]. However, KO shows some peculiar characteristics which differentiate it from the most commonly used FO. First, most of EPA and DHA contained in KO are esterified in the form of phospholipids, whereas in FO they are incorporated into triglycerides [6]. While the small intestinal lipid absorption is similar for both phospholipid and triglyceride forms, it has been proposed that they could influence tissue distribution [7]–[11]. Second, the ratio of EPA to DHA is higher in KO than in FO and third, KO is particularly rich in the antioxidant astaxanthin which increase its stability [12]. The beneficial effects of KO in the course of dyslipidemia and inflammation have been reported by several authors both in humans and in animals [13]–[16]. Furthermore, a higher potency of KO in comparison to FO in the modulation of the activity and the expression of many enzymes involved in lipid metabolism has been demonstrated [17], [18]. Nevertheless, there is still a need for further studies to reveal the molecular mechanisms behind the health-promoting effects of KO.

Hepatic lipogenesis, one of the anabolic pathways modulated by KO, is characterized by a complex series of reactions starting in the mitochondrial matrix and continuing in the cytosol. The excess of acetyl-CoA, produced in the mitochondrial matrix and deriving from the catabolic degradation of carbohydrates and amino acids, is at first incorporated into citrate, which is subsequently exported from mitochondria to the cytosol. The mitochondrial tricarboxylate carrier or citrate carrier (CIC) catalyzes the efflux of citrate, thereby connecting the catabolic pathways to the anabolic ones [19]. In fact, the transported citrate regenerates acetyl-CoA in the cytosol, which, in turn, is the primer not only for the *de novo* fatty acid synthesis but also for the cholesterol biosynthesis. Therefore, the CIC protein, along with the more investigated ACC and FAS, represents a good candidate for studies monitoring possible alterations in hepatic lipogenesis [20]–[22].

Previous studies have highlighted an involvement of the *de novo* fatty acid synthesis in the onset of hepatic steatosis [23], [24]. Interestingly, it has been reported that a dietary KO supplementation has the capacity to reduce fatty liver in mice [16]. Since we have recently found that KO is able to strongly suppress hepatic lipogenesis in animals fed with a standard diet [18], in this study we have investigated the molecular mechanisms underlying the possible protective effects of KO in animals fed a high-fat (HF) diet. To this end, we have thoroughly analyzed several enzymatic activities occurring in liver and belonging to both anabolic and catabolic pathways. In parallel, we have also monitored the changes in distinct metabolites during the selected dietary treatment. The obtained results led us to depict a possible framework for the molecular action of KO during this dysmetabolic condition.

## Results

### Effect of diets on food intake and body and liver weights

**Figure 1:**
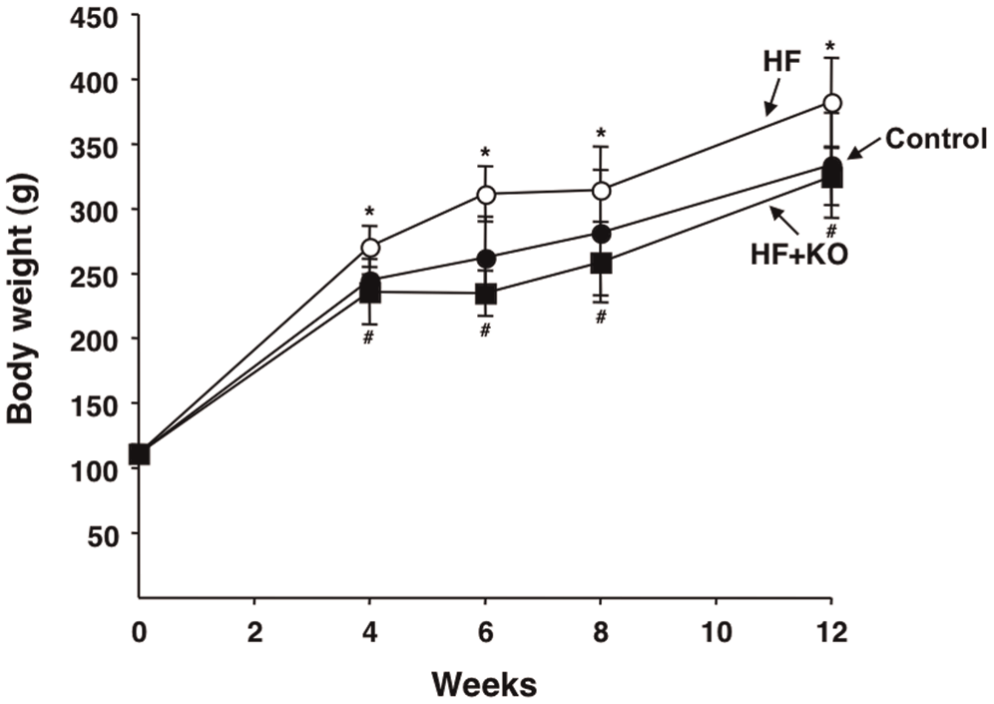
Effect of KO on body weight. Body weights of rats fed control (filled circle), HF (open circle) and HF+KO (filled square) diets are indicated for the treatment periods in weeks. Each point represents the mean ± SD for 10 animals. *P<0.05 *vs.* rats fed control diet; ^#^ *P*<0.05 *vs.* rats fed HF diet.

Animals (male Sprague-Dawley rats) were randomly divided into three groups and fed a control diet, a HF diet or a HF+KO diet for 12 weeks (Table 1). The food intake did not differ significantly between the three treatment groups during the study (Control group: 11.8±1.8 g/*die*; HF group: 12.4±0.7 g/*die*; HF+KO group: 11.8±1.4 g/*die*). On the contrary, a significant increase in body weight of rats belonging to the HF group was detectable already after 4 weeks of treatment, in comparison to control animals (Fig. 1). This finding was predictable on the basis of the higher caloric content of the HF diet with respect to the standard diet (Table 1). Interestingly, the supplementation of the HF diet with 2.5% KO (HF+KO group) significantly prevented this effect (Fig. 1). The liver weight did not differ significantly between the three groups at any time during the dietary treatment (data not shown).

**Table 1:** Composition of diets (%).

	Control	HF	HF+KO
**Proteins**	18.6	20.4	19.9
**Lipids**	6.2	35.2	36.8
Fatty acids			
14:0	-	0.5	0.6
16:0	0.7	8.7	9.0
18:0	0.2	4.3	4.3
16:1	-	-	0.1
18:1 (n9-7-5)	1.2	15.8	16.0
18:2 (n6)	3.1	3.5	3.5
18:3 (n3)	0.3	-	-
20:5 (n3) – EPA	-	-	0.3
22:6 (n3) – DHA	-	-	0.2
Σ SFA	0.9	13.5	13.9
Σ MUFA	1.3	15.8	16.1
Σ PUFA	3.4	3.5	4.1
Σ PUFA n-3	0.3	-	0.6
**Carbohydrates**	44.2	36.1	35.2
**kcal/100 g**	310	540	549

The control group of animals received a standard diet (Global Diet 2018S from Harlan Teklad). The HF group received a diet with 35% fat (Diet TD.03584 from Harlan Teklad) and the KO group was fed with the above reported HF diet supplemented with 2.5% KO. Fatty acids were extracted from the three diets and analyzed by gas-liquid chromatography.

### Hepatic *de novo* fatty acid synthesis

**Figure 2:**
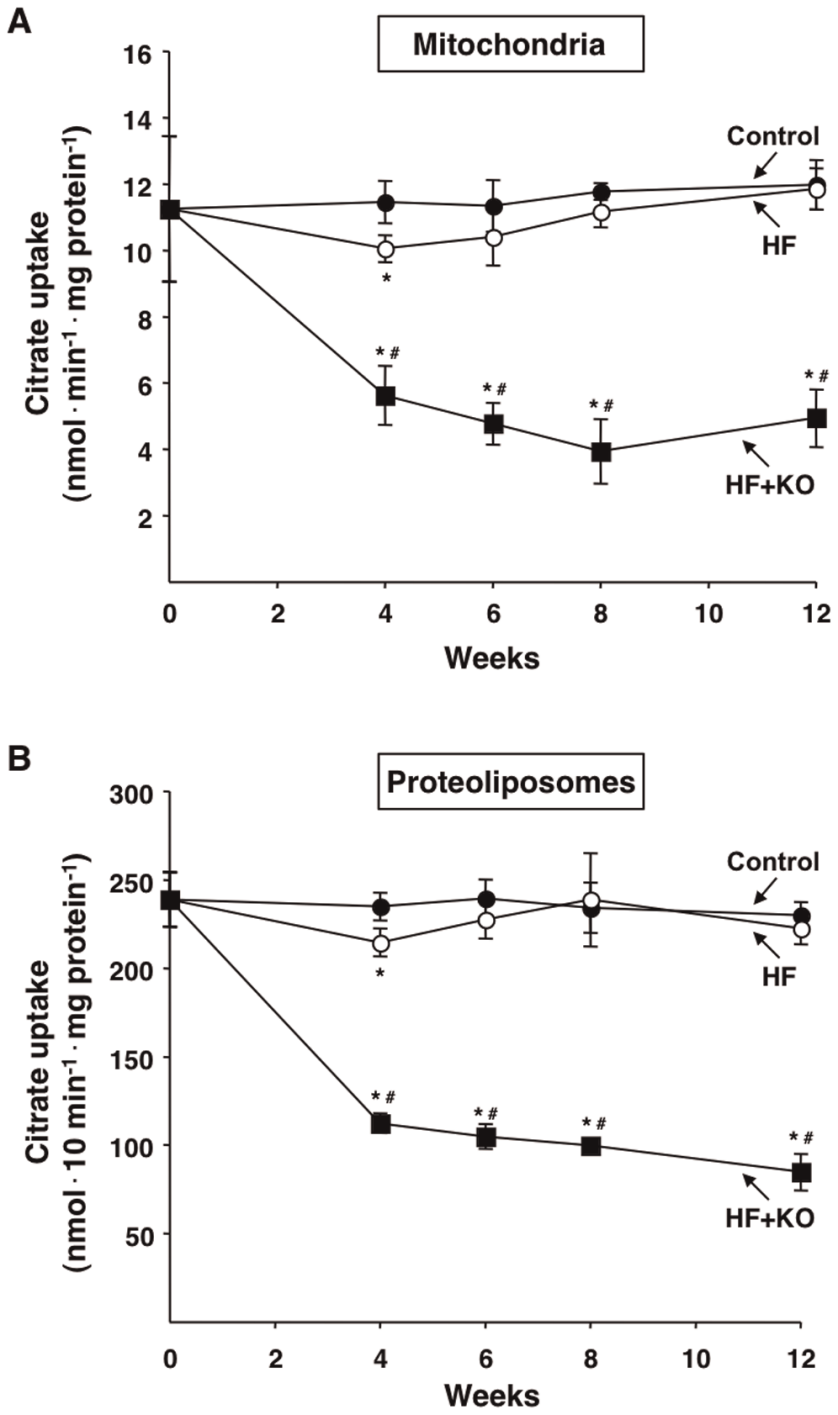
Effect of KO on the transport activity of mitochondrial CIC. Transport of citrate into rat liver mitochondria that are freshly isolated (A) and into a reconstituted system (proteoliposomes) (B) was measured at the times indicated. The values reported in the figure represent the means ± SD (*n* = 4). *P<0.05 *vs.* rats fed control diet; ^#^ *P*<0.05 *vs.* rats fed HF diet.

The anabolic pathway of fatty acid synthesis utilizes the carbon units transported outside liver mitochondria by the CIC [19]. For this reason, we investigated the transport activity of this protein in freshly isolated mitochondria from liver of the three groups of rats. The transport activity of this mitochondrial carrier remained almost unaffected over time in the control group (Fig. 2A). Approximately the same trend was observed in the case of the HF group, i.e. almost no influence of the HF diet on the CIC activity over time. A small yet significant inhibition of the CIC activity in the HF group (about 12%) was only found after 4 weeks of dietary treatment (Fig. 2A). A net and significant decrease in the CIC activity was instead found in mitochondria isolated from the HF+KO group. After 12 weeks, such a decrease was 59% in the HF+KO group with respect to both control and HF groups. In this context, it is important to underline that the CIC protein operates in the inner mitochondrial membrane where it is deeply embedded. Therefore, its transport activity can be influenced, at least in principle, by the phospholipid and fatty acid composition of the inner mitochondrial membrane. In order to investigate this possibility, we extracted the CIC protein from the mitochondrial membranes using a non-denaturing detergent and subsequently purified it by hydroxyapatite chromatography. Eventually, the purified CIC was functionally reconstituted into liposomes, thereby obtaining the so-called proteoliposomes. The transport activity of the purified and reconstituted CIC from the HF+KO animals was significantly lower over time in comparison to that of both control and HF groups (Fig. 2B). An inhibition of about 60% was found at the 12th week of dietary treatment in HF+KO animals with respect to the other two groups of animals. Interestingly, the activity of the mitochondrial CIC in the rats of the HF group, excluding the inhibition observed after 4 weeks, was practically identical to that of the control rats (Fig. 2B). These observations imply that the addition of 2.5% KO to the HF diet (35% fat) is able to significantly decrease the transport activity of the mitochondrial CIC.

**Figure 3:**
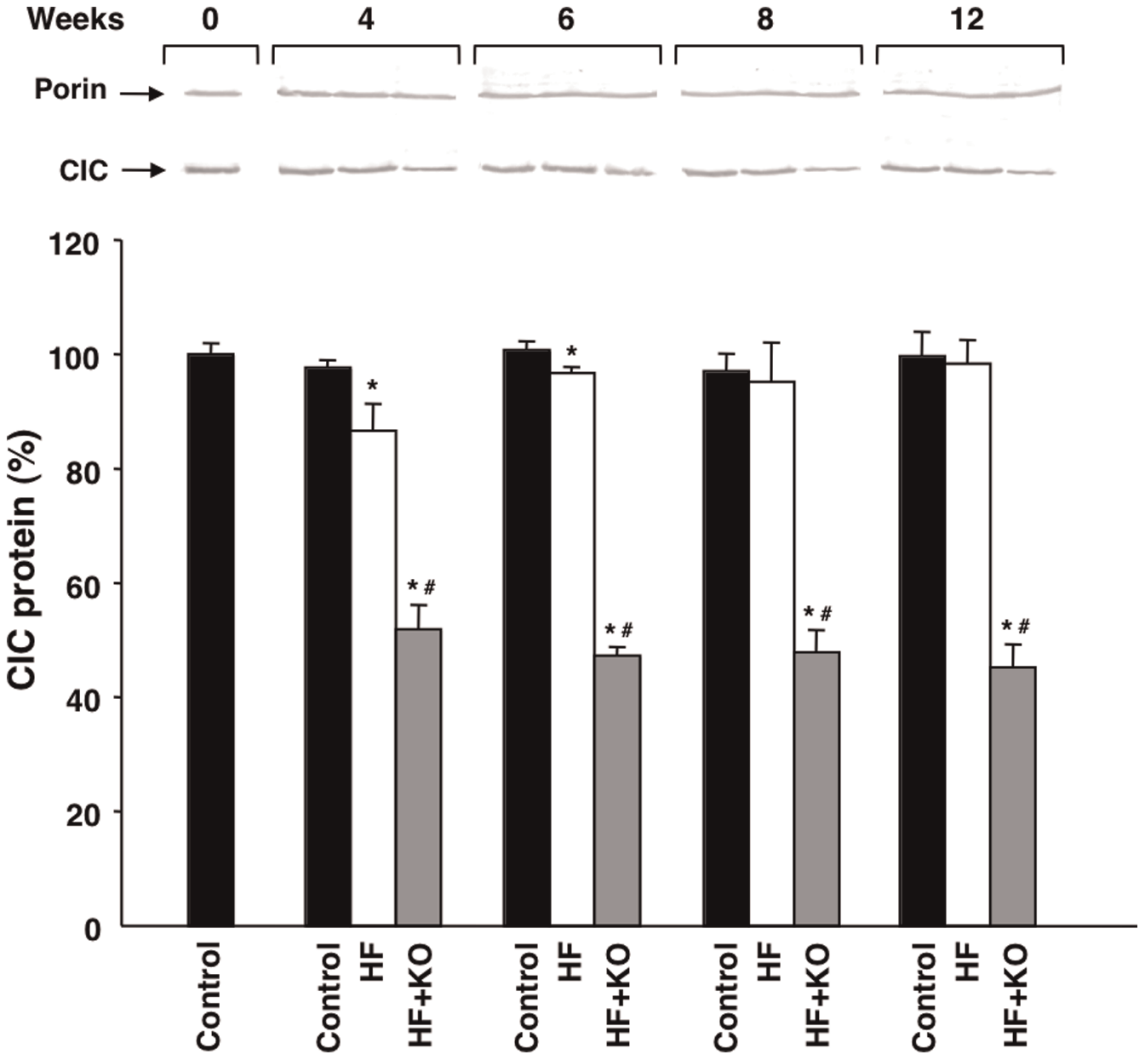
Effect of KO on protein levels of mitochondrial CIC. Liver mitochondrial proteins from control, HF or HF+KO-fed rats were separated by SDS-PAGE, transferred to nitrocellulose and then immunodecorated with antisera against either the rat CIC or the mammalian porin. The values reported in the graph represent the means ± SD (*n* = 4; *P<0.05 *vs.* rats fed control diet; ^#^ *P*<0.05 *vs.* rats fed HF diet). The amount of CIC revealed by immunodecoration at the beginning of dietary treatment was set to 100%.

Furthermore, the kinetic properties of the CIC activity were investigated in the proteoliposomal system. No significant difference was found in the Km values for the reconstituted CIC activity in the three groups of rats (Table 2). On the contrary, a net decrease in the Vmax values was found in the HF+KO group with respect to both the control and the HF group. In good agreement with the above mentioned results (Fig. 2A and B), a similar extent of inhibition (about 67%) was found in the case of Vmax after 12 weeks of dietary treatment. These findings were fully validated by western blot experiments in which the expression of the mitochondrial CIC was monitored over time in the three treatment groups. The decrease in the CIC activity found in the HF+KO rats was accompanied by a strong decrease in the amount of the mitochondrial carrier protein in the same group of animals (Fig. 3). After 12 weeks of HF+KO dietary treatment the amount of mitochondrial CIC decreased 55%, compared to control and HF groups. Interestingly, a small, although significant, decrease in the amount of CIC protein was also found at the 4th week in the HF group in comparison to the control group. These results suggest that the CIC inhibition due to KO supplementation of the HF diet depends on a strong decrease in the expression of this mitochondrial carrier protein. The amount of porin, an outer membrane protein tested as a control, did not change in any group at any time of treatment.

**Table 2:** Km and Vmax of citrate transport in a reconstituted system.

Weeks	Km (mM)			Vmax (nmol·min^−1^·mg protein^−1^)		
	Control	HF	HF+KO	Control	HF	HF+KO
0	0.187	-	-	143.3	-	-
4	0.199	0.184	0.205	141.0	124.0	70.5
6	0.189	0.190	0.203	141.6	133.5	64.4
8	0.189	0.189	0.190	142.0	142.0	57.1
12	0.187	0.187	0.188	141.3	143.1	46.3

Km and Vmax values were measured in a reconstituted system at the times indicated. Proteoliposomes were reconstituted with the CIC as described in the Methods section. [^l4^C] Citrate, 0.04–0.40 mM, was added to proteoliposomes containing 10 mM citrate. The citrate/citrate exchange was stopped 1 min after the addition of the radiolabeled substrate by 20 mM 1,2,3-BTA. Km and Vmax values were calculated by linear regression.

**Figure 4:**
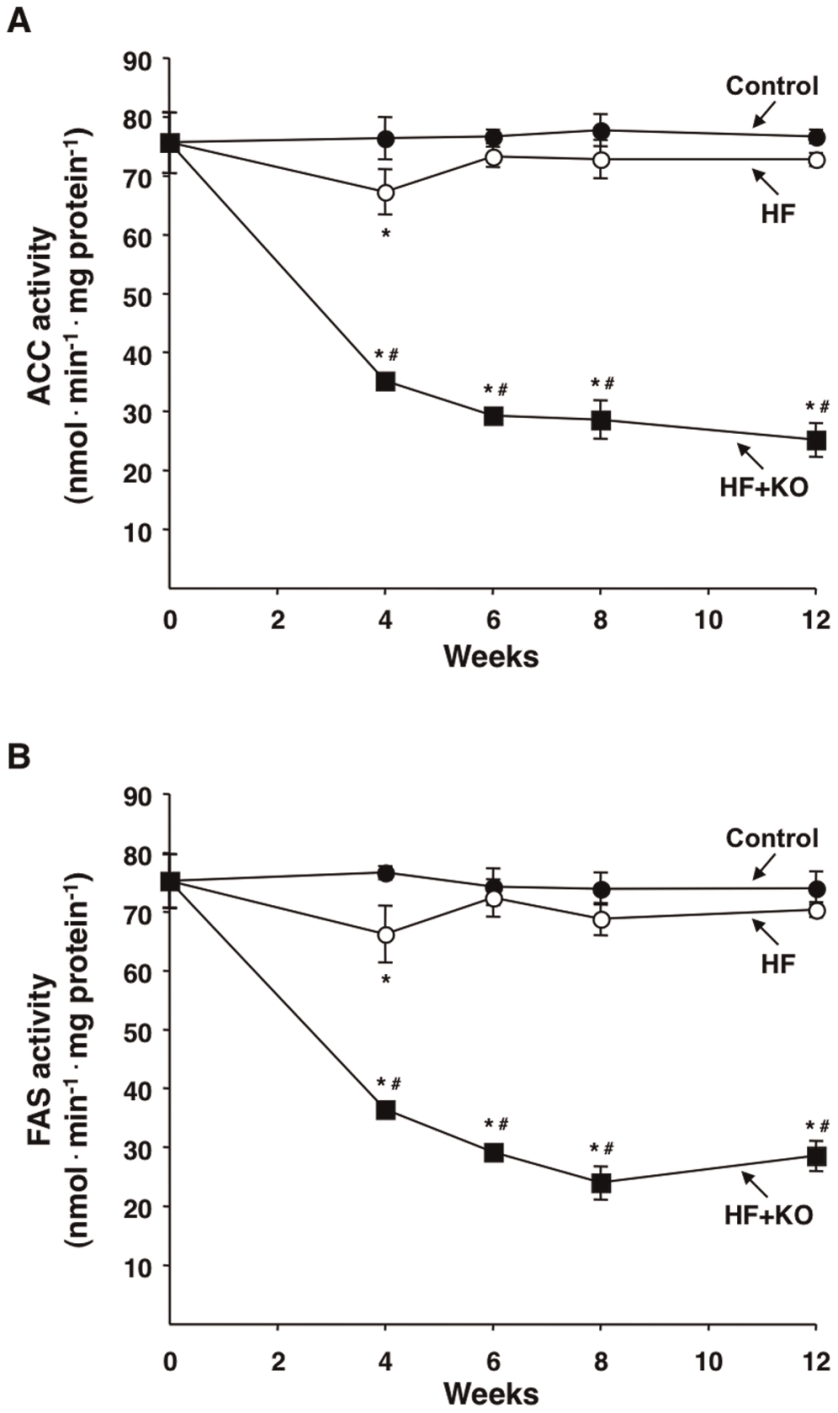
Effect of KO on lipogenic enzyme activities. The activities of ACC (A) and FAS (B) were measured in the cytosol of rat hepatocytes at the times indicated. The values are expressed as nanomoles of NADH (ACC) or NADPH (FAS) oxidized min^−1^ mg protein^−1^ and represent the means ± SD (*n* = 4). *P<0.05 *vs.* rats fed control diet; ^#^ *P*<0.05 *vs.* rats fed HF diet.

The activities of two cytosolic enzymes, ACC and FAS, to which the mitochondrial CIC supplies carbon units for hepatic fatty acid synthesis, were also investigated. A net decrease of the ACC activity in the HF+KO group was observed, when compared to that of both HF and control groups (Fig. 4A). After 12 weeks, the ACC activity in the HF+KO group was reduced by about 65%, when compared to control animals. A similar behaviour was observed in the case of the FAS activity (Fig. 4B), with approximately 60% inhibition in the HF+KO group. Hence, parallel inhibitions were found in the activities of the cytosolic ACC and FAS and of the mitochondrial CIC.

### Hepatic fatty acid oxidation

**Figure 5:**
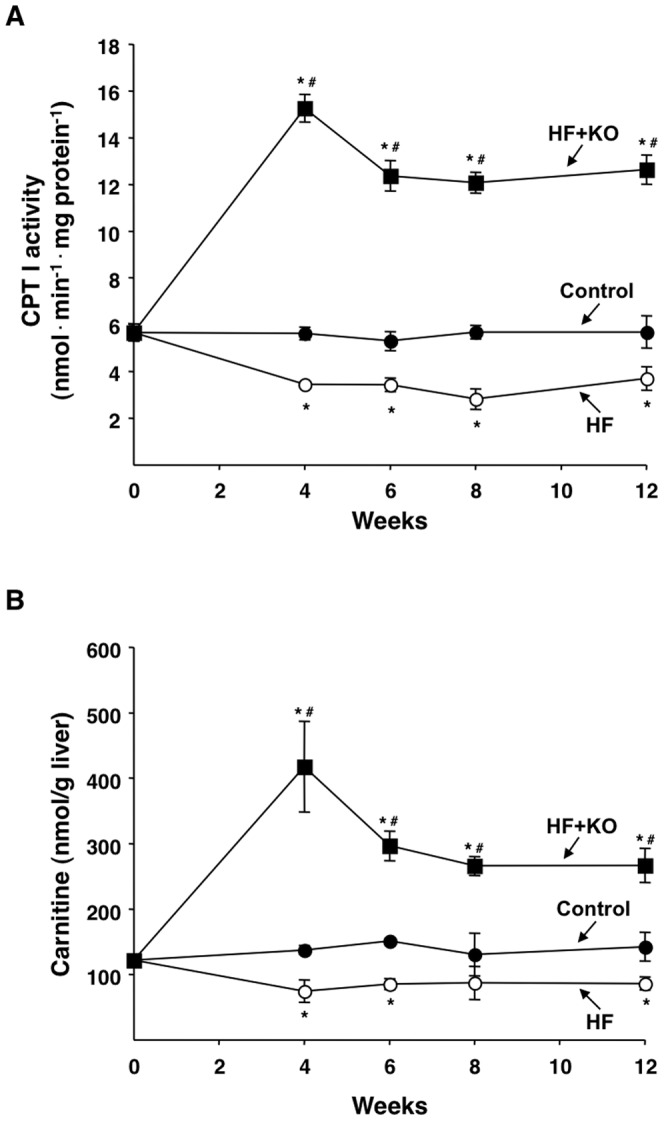
Effect of KO on hepatic fatty acid oxidation. (A) CPT I activity was measured in liver mitochondria freshly isolated from rats at the times indicated. The values are expressed as nanomoles of DTNB reduced min^−1^ **·**mg protein^−1^ and were calculated as described in the Methods section. (B) Liver carnitine levels were also determined at the times indicated. Data are means ± SD (*n* = 4). *P<0.05 *vs.* rats fed control diet; ^#^ *P*<0.05 *vs.* rats fed HF diet.

The catabolic pathway of fatty acid oxidation occurs inside mitochondria and, from a metabolic point of view, represents the opposite of fatty acid synthesis. The rate-limiting step of fatty acid oxidation is represented by the activity of CPT I which is involved in the transport of fatty acids into the mitochondrial matrix. The activity of CPT I remained practically constant over time in the control rats (Fig. 5A). However, in the HF animals a clear decrease in the CPT I activity was detected at any time during the dietary treatment. The maximum degree of inhibition (51%) was seen after 8 weeks of dietary tratment. On the contrary, a significant increase in CPT I activity was found in the HF+KO rats. After 12 weeks of feeding, the CPT I activity was 2.1 and 3.4 fold higher than those of control and HF rats, respectively. Notably, these results were substantiated by the levels of hepatic carnitine detected in the three groups of animals (Fig. 5B). Carnitine is coupled to fatty acids in order to facilitate their passage across the inner mitochondrial membrane. In fact, the carnitine levels paralleled the trend of CPT I activities found in control, HF and HF+KO rats (Fig. 5B). This is in line with the fact that an increase (or a decrease) in CPT I activity is accompanied by parallel changes in free carnitine levels [25]. Thus, KO supplemented to the HF diet strongly stimulates fatty acid oxidation.

### Hepatic mitochondrial oxidative phosphorylation

An increased fatty acid oxidation supplies higher levels of reducing equivalents, which are normally addressed towards the mitochondrial oxidative phosphorylation for ATP production. We therefore analyzed the respiratory efficiency of freshly isolated mitochondria by oxygraphic methods (Table 3). V_3_, also known as the active state of respiration (measured in the presence of externally added ADP and respiratory substrates), did not show massive changes in any of the treatment groups. In the rats fed the HF diet a little yet significant decrease in V_3_ was observed from the 6th week of dietary treatment onward, in comparison to control animals. In the HF+KO animals, less evident changes in the V_3_ values were found. V_4_, also known as the resting state of respiration (measured in the presence of respiratory substrates when added ADP had been completely phosphorylated to ATP), showed more differences (Table 3). Whereas the V_4_ values in the HF+KO group were practically identical to those found in control animals, except the value measured at the 4th week, the HF treatment led to a significant increase in V_4_ values at any time measurement. After 12 weeks, the V_4_ measured in HF treated animals was about 2.6 fold higher than those found in control and HF+KO groups. As a consequence, the RCR (respiratory control ratio) values were profoundly and significantly lower in HF animals with respect to those calculated for both control and HF+KO animals. The RCR is a direct measure of the mitochondrial respiratory efficiency [26], [27] and the values found in animals fed the HF diet suggest that the excess of dietary fat most probably is the cause of a partial uncoupling between respiration and phosphorylation in mitochondria. Interestingly, the dietary KO supplementation is able to efficiently abolish this effect, keeping the mitochondrial respiratory efficiency unaltered (Table 3).

**Table 3:** Mitochondrial respiratory efficiency.

Weeks	V_3_ (nmol O_2_ ml^−1^ min^−1^)			V_4_ (nmol O_2_ ml^−1^ min^−1^)			RCR		
	Control	HF	HF+KO	Control	HF	HF+KO	Control	HF	HF+KO
0	73.2±5.9	-	-	11.4±0.8	-	-	6.4±0.4	-	-
4	65.2±6.9	61.9±4.9	88.4±3.2* #	10.1±2.3	27.9±2.7*	24.0±1.2* #	6.6±1.0	2.2±0.2*	4.1±0.3#
6	76.8±9.9	54.8±1.3*	58.9±5.5* #	13.1±1.5	26.6±3.3*	10.0±1.0#	5.9±1.2	2.1±0.3*	5.9±0.2* #
8	83.4±8.9	64.8±3.7*	79.2±4.1#	15.3±3.1	32.8±1.4*	14.7±3.1#	5.6±1.6	2.0±0.1*	5.4±0.8#
12	76.8±6.1	61.9±7.0*	69.5±4.1	12.0±0.7	29.9±0.9*	10.8±1.8#	6.4±0.6	2.1±0.2*	6.5±1.2#

Respiratory control ratio (RCR) was calculated as the ratios of the rate of oxygen uptake in the presence of added ADP (V_3_) to the rate observed when added ADP had been completely phosphorylated to ATP (V_4_).

* *P*<0.05 *vs.* rats fed control diet;

# *P*<0.05 *vs.* rats fed HF diet; *n* = 3.

**Figure 6:**
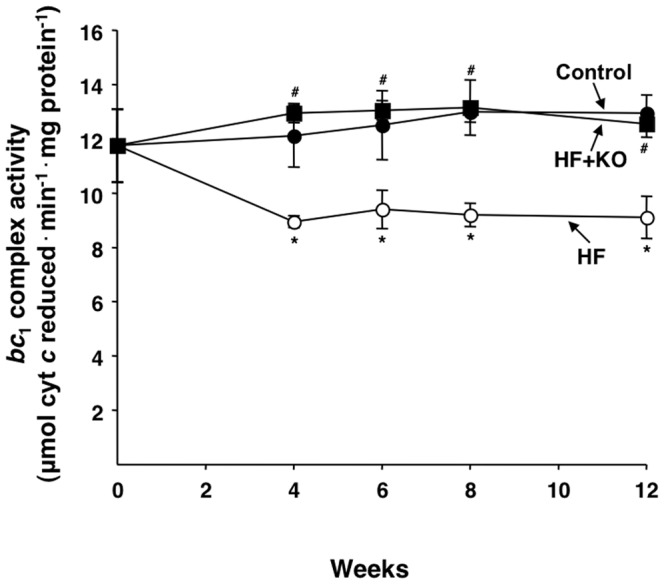
Effect of KO on mitochondrial *bc*_1_ complex activity. The activity of complex III of the respiratory chain was measured in liver mitochondria freshly isolated from rats at the times indicated. The values are expressed as micromoles of cytochrome *c* reduced min^−1^·mg protein^−1^ and represent the means ± SD (*n* = 4). *P<0.05 *vs.* rats fed control diet; ^#^ *P*<0.05 *vs.* rats fed HF diet.

In a more selective approach for investigating the functionality of the mitochondrial oxidative phosphorylation we assayed the activity of a single component of the respiratory chain, the cytochrome *bc*_1_ complex or complex III [28]. A significant decrease in the activity of the cytochrome *bc*_1_ complex was found in HF animals at any time measurement (Fig. 6). After 12 weeks of treatment, the decrease in mitochondrial *bc*_1_ activity was about 30% compared to control rats. On the contrary, the values of the cytochrome *bc*_1_ activity of the HF+KO rats were practically identical to those measured in control animals (Fig. 6).

### Hepatic lipid and protein oxidation

**Figure 7:**
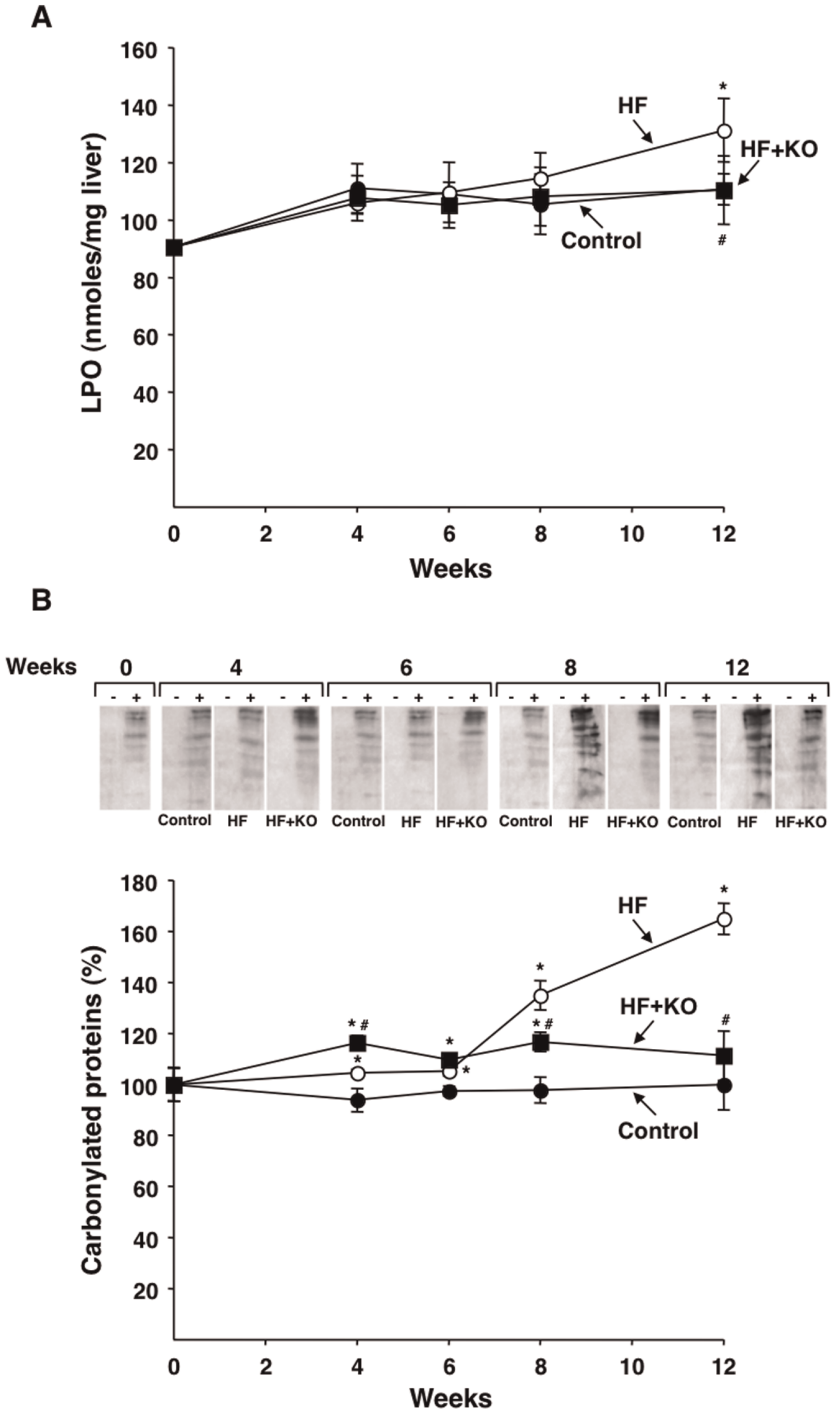
Effect of KO on oxidative modification of protein and lipid. (A) Liver lipid peroxide (LPO) levels were determined at the times indicated. Each point represents the mean ± SD for 4 liver samples. (B) DNP-derivatized liver tissue lysates (lanes+) from control, HF or HF+KO-fed rats were analysed for the presence of oxidized protein. DNP protein bands were visualized by chemiluminescence. Oxyblot images were analyzed by densitometry and the values reported in the graph represent the means ± SD (*n* = 4). The amount of carbonylated proteins revealed at the beginning of dietary treatment was set to 100%. *P<0.05 *vs.* rats fed control diet; ^#^ *P*<0.05 *vs.* rats fed HF diet.

It has been reported that a dysfunction of the mitochondrial oxidative phosphorylation may represent one of the causes responsible for the increase of reactive oxygen species (ROS) [29]. ROS, in turn, may oxidatively modify cellular protein and lipid, thereby leading to the appearance of several pathologies. The oxidative damage of hepatic lipids is shown in Fig. 7A. Whereas the lipid peroxide (LPO) levels were identical in both control and HF+KO rats at any time, a significant increase (+19%) was found in the HF animals after 12 weeks of treatment. The analysis of protein oxidation in liver (Fig. 7B) revealed a strong increase in the level of oxidized proteins in HF animals at 8 (+38%) and 12 (+65%) weeks of dietary treatment (Fig. 7B). On the contrary, the values found in HF+KO rats at the 4th, 6th, and 8th week were only slightly increased in comparison to those measured in control animals. At the 12th week of dietary treatment no significant difference in the oxidative modification of protein was detected between the HF+KO and the control group of animals (Fig. 7B). It appears, therefore, that the HF diet is able to increase the oxidative damage of both proteins and lipids, expecially at longer feeding periods, and that this effect is efficiently reversed by KO supplementation of the HF diet.

### Plasma and liver lipids

**Figure 8:**
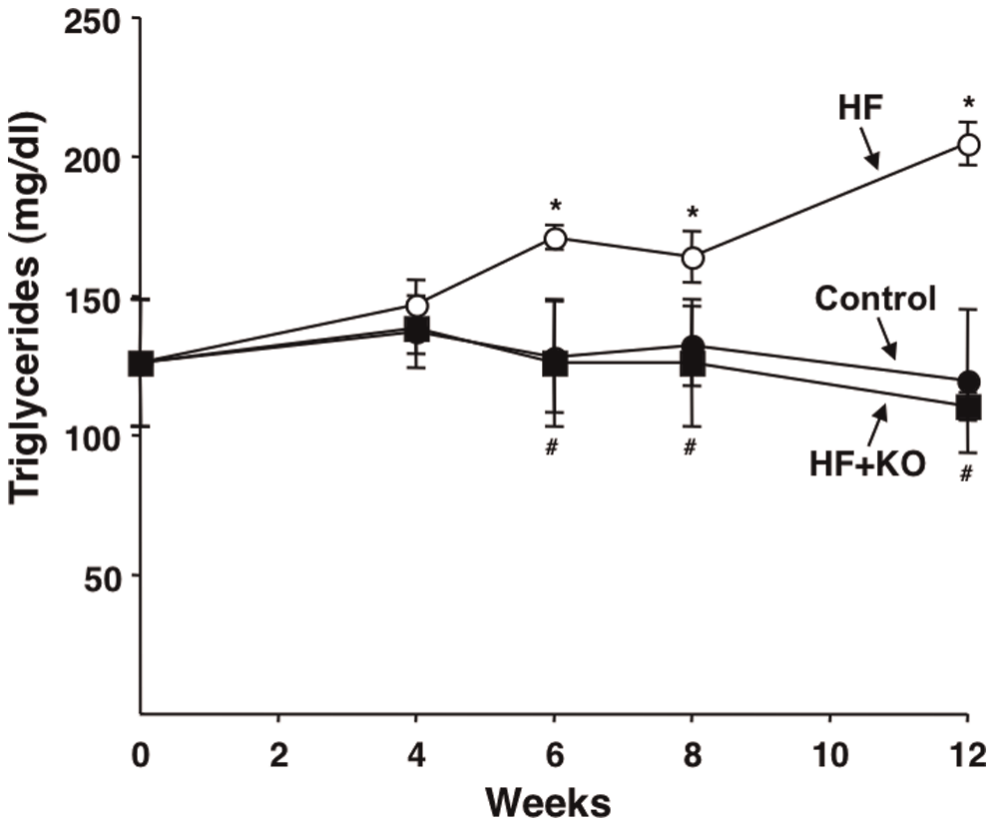
Effect of KO on plasma triglycerides. The levels of plasma triglycerides were determined at the times indicated, using commercial kits. The values reported in the figure represent the means ± SD (*n* = 4). **P<0.05 *vs.* rats fed control diet; ^#^ *P*<0.05 *vs.* rats fed HF diet.

A significant increase in the level of plasma triglycerides was found in the HF rats starting from the 6th week (Fig. 8). At the 12th weeks, an increase in the level of plasma triglycerides of 71% was found in the HF rats, compared to control animals (Fig. 8). On the contrary, no significant difference was detected in the triglyceride levels between control and HF+KO animals. The subsequent assay of the levels of plasma cholesterol and phospholipids did not reveal any significant difference between the three groups at any time of dietary treatment (data not shown).

**Figure 9:**
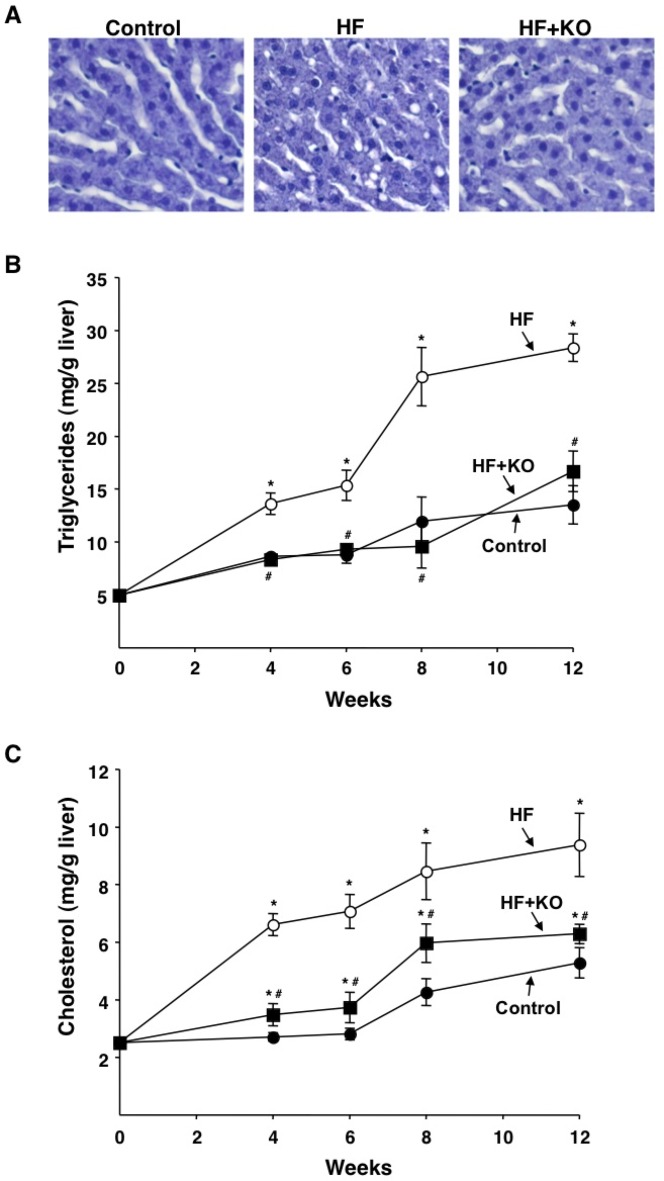
Effect of KO on liver lipids. (A) Cresyl violet staining of liver histological sections from rats fed for 12 weeks with control, HF or HF+KO diet. The levels of liver triglycerides (B) and cholesterol (C) were determined at the times indicated. Each point represents the mean ± SD for 4 liver samples. *P<0.05 *vs.* rats fed control diet; ^#^ *P*<0.05 *vs.* rats fed HF diet.

Additionally, there were differences in lipid levels found in liver. The liver histologic examination revealed microvesicular fat depositions in HF rats, whereas no fat deposition was found in the HF+KO animals (Fig. 9A). After 12 weeks, the liver triglyceride content was 2.1 fold higher, compared to that in control animals. Interestingly, the KO supplementation of the HF diet reversed this effect, thereby assuring liver triglyceride levels very similar to those of control rats (Fig. 9B). Also the levels of total cholesterol significantly increased in HF rats in comparison to those of control animals (+77% after 12 weeks) (Fig. 9C). In the HF+KO animals, instead, the increase in cholesterol levels was significantly less evident being about +19% at the 12th week (Fig. 9C). The assay of liver phospholipid content did not reveal any significant difference among the various groups of animals at any time (data not shown). Accordingly, the dietary administration of KO in animals fed a HF diet has a normalizing effect on the hepatic content of both triglycerides and cholesterol.

### Lipid analysis of mitochondrial membranes

**Figure 10:**
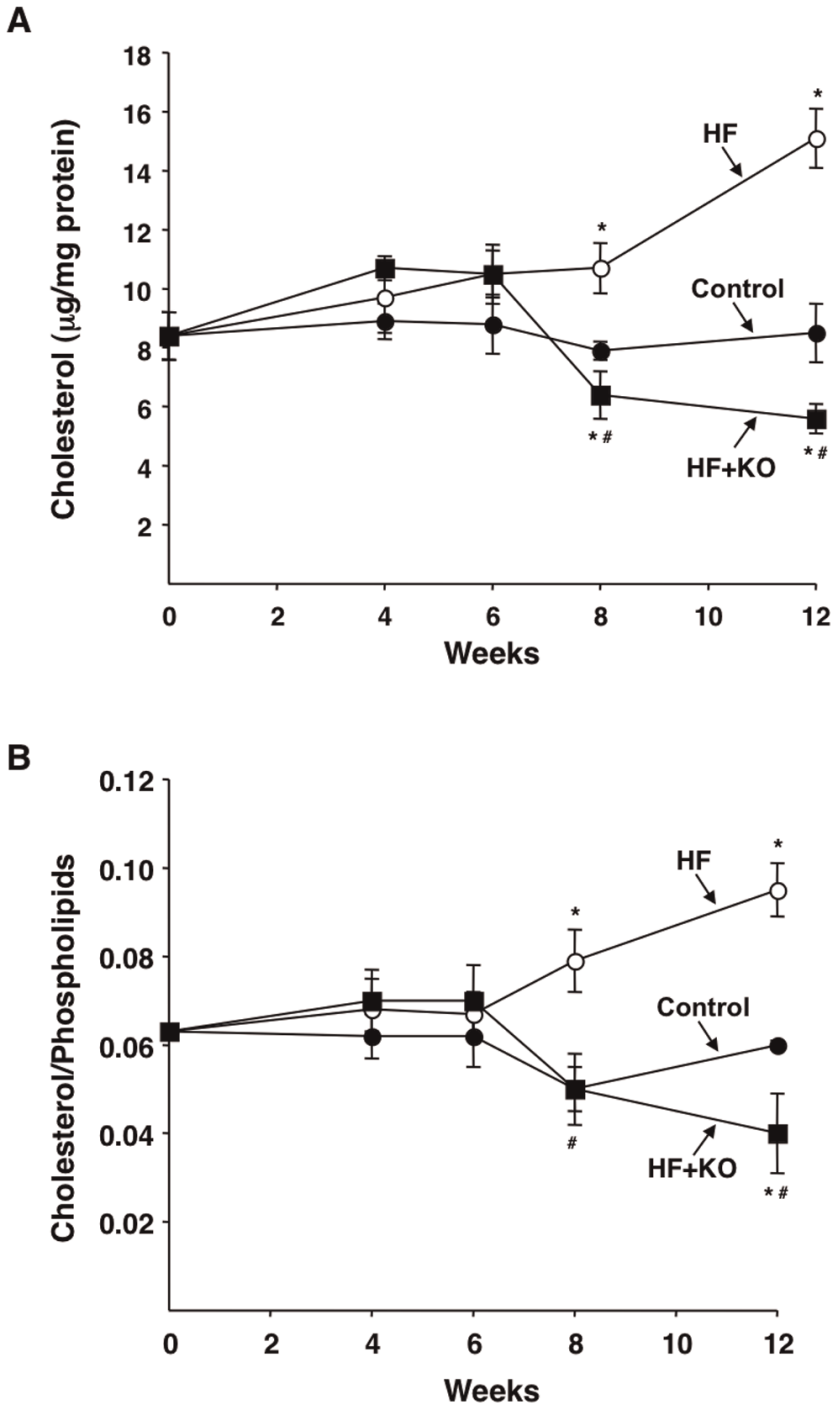
Effect of KO on cholesterol and phospholipid contents in liver mitochondria. The levels of liver cholesterol (A) and cholesterol/phospholipids ratio (B) were determined at the times indicated. Each point represents the mean ± SD (*n* = 3). *P<0.05 *vs.* rats fed control diet; ^#^ *P*<0.05 *vs.* rats fed HF diet.

Cholesterol and phospholipid contents of mitochondrial membranes from control and treated animals were analyzed. No significant difference was observed in the phospholipid concentration and composition of hepatic rat mitochondria in the three groups (data not shown). However, after 8 weeks of treatment, cholesterol content of mitochondrial membranes was affected upon HF and HF+KO treatments. There was a strong increase in the level of cholesterol in HF animals after 8 (+35%) and 12 (+78%) weeks of dietary treatment, in comparison to the values found in control animals (Fig. 10A). On the contrary, the values found in HF+KO rats were lower than those of the control group (20% and 34% decrease after 8 and 12 weeks, respectively). Accordingly, the cholesterol/phospholipid ratio was significantly higher in the liver mitochondria from HF rats in comparison to the control, whereas a strong reduction in this parameter was observed in the HF+KO group (Fig. 10B).

Mitochondrial fatty acid composition was noticeably different among the three treatment groups (Table 4). Palmitic acid (16:0) and stearic acid (18:0) increased, whereas linoleic acid (18:2) decreased in mitochondria from HF and HF+KO rats. Interestingly, DHA (22:6) strongly increased in the HF+KO group, thus leading to an increase of the fatty acid unsaturation index (U.I.) of about 25% in comparison to that of the HF rats after 12 weeks of dietary treatment. It appears, therefore, that KO supplemented to the HF diet is able to influence mitochondrial membrane fluidity, by modifying both cholesterol content and fatty acid composition.

**Table 4:** Fatty acid composition (mol%) of mitochondrial membrane phospholipids.

		14:0	16:0	16:1	18:0	18:1	18:2	18:3	20:4	20:5	22:6	U.I.
	**Control**	0.6	22.0	0.5	23.0	7.4	20.0	ND	12.0	0.2	2.7	**113**
**Week 0**	**HF**	-	-	-	-	-	-	-	-	-	-	**-**
	**HF+KO**	-	-	-	-	-	-	-	-	-	-	**-**
	**Control**	0.6	24.0	0.4	25.0	9.5	21.0	ND	15.0	0.4	2.5	**129**
**Week 4**	**HF**	0.8	27.0	0.3	29.0	13.0	11.2	ND	13.0	0.2	2.5	**99**
	**HF+KO**	0.3	30.0	0.4	28.0	12.0	9.9	ND	9.0	0.4	4.5	**97**
	**Control**	0.6	24.0	0.4	25.0	13.0	19.8	ND	15.0	0.3	2.7	**131**
**Week 6**	**HF**	0.7	28.0	0.1	29.0	13.0	11.0	0.3	15.0	ND	2.9	**113**
	**HF+KO**	0.4	29.0	0.3	29.0	11.0	13.0	0.4	12.3	0.6	5.4	**123**
	**Control**	0.5	21.0	0.3	26.0	8.3	18.9	0.1	11.0	ND	2.2	**103**
**Week 8**	**HF**	0.3	30.0	0.1	31.0	12.5	8.6	ND	13.5	0.5	2.5	**86**
	**HF+KO**	0.3	28.0	0.4	32.0	9.6	10.4	0.2	12.1	0.1	4.3	**106**
	**Control**	0.6	25.0	0.4	23.0	9.0	20.0	0.1	13.6	ND	2.7	**120**
**Week 12**	**HF**	0.4	27.0	0.2	29.9	14.8	9.1	ND	11.4	0.2	2.5	**95**
	**HF+KO**	0.5	35.0	0.4	21.0	9.2	10.0	0.2	13.0	0.1	6.1	**119**

Data correspond to the mean of values obtained in two independent preparations. Differences between values of every couple were less than 10%. Fatty acids were extracted from mitochondrial membrane phospholipids after saponification. After derivatization with methanolic boron trifluoride, fatty acid methyl esters were separated by gas-liquid chromatography and identified by using known standards.

U.I., Unsaturation index, Σ mol% of each fatty acid x number of double bonds of the same fatty acid; ND, not detected.

### Plasma levels of glucose and insulin

**Figure 11:**
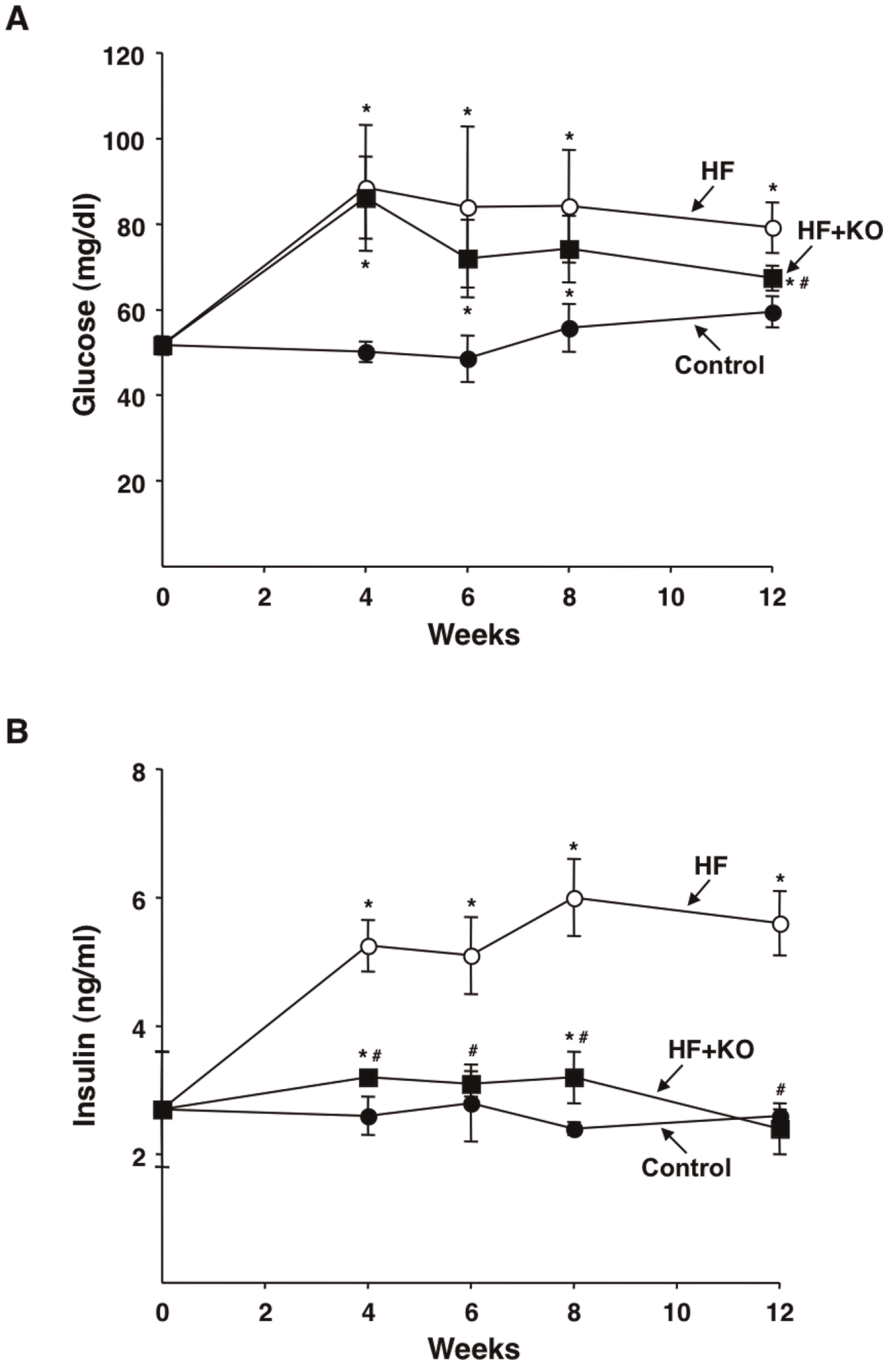
Effect of KO on plasma levels of glucose and insulin. (A) Blood glucose concentrations were determined using reactive strips and a commercial glucometer. (B) Plasma insulin concentrations were analyzed with commercial enzyme-linked immunosorbent assay kits. The values reported in the figure represent the means ± SD (*n* = 4). *P<0.05 *vs.* rats fed control diet; ^#^ *P*<0.05 *vs.* rats fed HF diet.

The blood glucose concentration increased in both HF (76%) and HF+KO (72%) animals after 4 weeks of dietary administration, in comparison to control rats (Fig. 11A). From the 4th week onward, there was a progressive normalization in the levels of blood glucose in the HF+KO treated animals, which resulted in only slightly increased level after 12 weeks (13%), compared to control rats. On the contrary, the modifications in glucose level were more persistent in the HF treated animals, compared to the control group (+33% at the 12th week). A sudden and massive increase in the levels of insulin was revealed in the plasma of HF animals, in comparison to control rats (Fig. 11B). After 12 weeks of dietary treatment, insulin was increased 2.15 fold in HF animals, in comparison to the control ones. However, the concentration of plasma insulin in rats supplemented with KO was comparable to that of control animals at any time during the dietary treatment (Fig. 11B). KO is therefore able to efficiently counteract the hyperglycidemic effects of a HF diet by normalizing the blood glucose level and preventing an increase in the plasma insulin concentration.

## Discussion

The results reported in this study suggest that KO has a strong capability to suppress the hepatic steatosis induced by a HF diet administered to rats. Indeed, the addition of a low amount of KO (2.5%) to a diet containing a large excess of fat (35%) efficiently prevented the accumulation of triglycerides and cholesterol inside rat hepatocytes. In agreement with these results, lower levels of triglycerides were also found in plasma of HF+KO animals in comparison to the values detected in HF rats. Overall, KO was able to keep the lipid concentrations similar or even identical to those found in control animals fed a standard diet containing 6% fat. This is of significance for the use of KO as a novel dietary source of EPA and DHA in preventing cardiovascular diseases.

Nevertheless, it is even more interesting to unveil the molecular mechanisms responsible for these beneficial effects. Indeed, the pathogenesis of metabolic syndrome and/or cardiovascular diseases appears quite complex and recent reports suggest intricate networks of metabolic modifications occurring in distinct parts of the organism mediated by several and partially unknown signals [30]. It is therefore difficult to depict a general scheme including all the molecular steps influenced by an ingested nutrient in the context of the entire organism. For this reason we have focused our attention on the molecular modifications induced by KO in liver that plays a central role in catabolic and anabolic pathways of lipid metabolism.

The results obtained indicate that the supplementation of KO to a HF diet strongly inhibits the hepatic fatty acid synthesis by reducing the activity and the expression of the mitochondrial CIC. The KO-induced CIC inhibition decreases the efflux of mitochondrial citrate towards the cytosol, thereby exerting at least a dual effect. On one hand, there is a reduced supply of carbon units in the cytosol and this in turn decreases the amount of substrate available for hepatic fatty acid synthesis. On the other hand, there is less citrate available for the stimulation of the cytosolic ACC. These combined actions significantly and negatively influence hepatic lipogenesis. In parallel to the inhibition of CIC activity, the dietary KO supplementation also induces a strong decrease in the hepatic ACC and FAS activities. These findings therefore suggest that there is a concerted reduction of the activity of key enzymes involved in hepatic lipogenesis. Such an effect was also observed in animals fed with a standard diet supplemented with the same low concentration of KO [18]. It appears therefore that KO has an intrinsic capability of reducing hepatic lipogenesis and that the potency of this effect is not lost in the presence of a big amount of fat in the diet, as reported in this study. Concomitantly, there is a strong increase in fatty acid oxidation found in the liver of HF+KO animals. This metabolic modification most probably can be explained by the lower levels of malonyl-CoA, the product of ACC activity. In fact, high levels of malonyl-CoA inhibit CPT I activity which, in this case, is instead strongly stimulated.

Besides the stimulation of fatty acid oxidation, KO also influences the mitochondrial respiratory efficiency. Indeed, in the HF rats a strong decrease in the mitochondrial respiratory efficiency was clearly detected and this effect was due to a possible uncoupling effect exerted by the excess of fatty acids present in this kind of diet [31], [32]. Interestingly, the addition of KO to the HF diet almost completely reversed this effect, thereby leading to RCR values comparable to those of control rats. Overall, this means that KO is able to induce the burning of excess of fat introduced by a hypercaloric diet, hence preventing the onset of fatty liver and at the same time leading to a reduction in body weight. These effects mainly, even if not exclusively, derive from a combination of distinct molecular mechanisms, such as: i) stimulation of fatty acid oxidation, ii) retention of normal mitochondrial respiration efficiency, and iii) inhibition of *de novo* lipogenesis.

A similar study, i.e the analysis of the effects of a KO-supplemented diet, has recently been carried out in HF fed mice [16]. While in the present study a KO-dependent reduction of body weight of HF fed rats was observed (Fig. 1), such an effect was not visible in HF fed mice under KO treatment [16]. On the contrary, in the study of Tandy *et al.* 2009 an increase in liver weight was clearly visible in HF fed mice and this effect was partly reversed by KO supplementation. Although we did not find any significant increase in liver weight of HF fed rats in the present study, the increase in the body weight observed in these animals would suggest a peripheral fat deposition. However, the addition of KO to the HF diet was able to efficiently reverse the triglyceride accumulation found in the liver of these animals (Fig. 9A). Hence, the results reported in this study further strengthen the ability possessed by KO to reduce liver lipid accumulation, even when there is no evident increase in liver weight. Various possible explanations exist for the differences found in these studies, such as the different animal species, the different dietary treatments, length of study and so on. It is however clear that the main effects exerted by KO supplementation to a HF diet, such as reduction of hepatic steatosis and improvement of lipid metabolism, are similar in both these studies.

Moreover, in the present study, KO was able to influence the membrane lipid composition of liver mitochondria by reducing cholesterol content and by increasing DHA levels. An increase in the liver levels of DHA, which has a structural role (whereas EPA is preferentially utilized for fatty acid oxidation or eicosanoid synthesis) [33] was also observed by Tou *et al.* [34]. We can however exclude that these modifications were in some way responsible for the observed reduced transport activity of the mitochondrial CIC (Fig. 2A). Indeed, a similar degree of inhibition was found after reconstitution of the CIC activity into liposomes which, differently from the mitochondrial membranes, showed a well defined and constant lipid composition (Fig. 2B).

Noteworthy is also the effect of KO on the levels of haematic glucose and insulin. An excess of dietary fat is often accompanied by an increase in glycemia because of a reduced utilization of sugar by peripheric tissues [35], [36]. In accordance with this, an increase of plasma insulin is also commonly found in HF fed animals indicating a possible insulin resistance by extra-hepatic tissues [37], [38]. Interestingly, the supplementation of KO to the HF diet reversed the hyperglycemic effect in a time-dependent manner and blocked any increase in the level of circulating insulin. This finding is in agreement with the prevention and/or reversal of insulin resistance exerted by dietary EPA and DHA [39]. It is however puzzling the finding of elevated, even if progressively decreasing, glucose levels in HF+KO animals at the 4th, 6th and 8th weeks despite the low insulin levels found at the same time intervals. This phenomenon is currently unexplained and merits further investigation.

**Figure 12:**
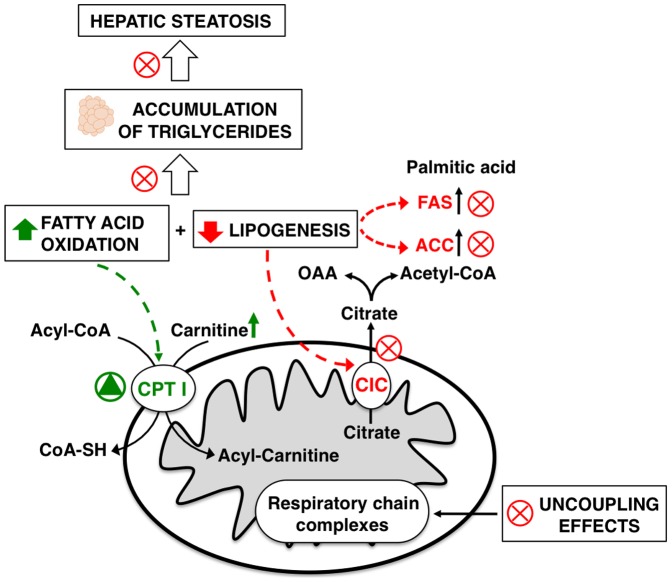
Liver metabolic pathways influenced by the addition of KO to a HF diet. A red X symbolizes inhibition; a green triangle symbolizes stimulation. OAA, oxaloacetate.

Fig. 12 depicts, in a schematic way, all the above described liver metabolic pathways influenced by the addition of KO to a HF diet. It became evident that KO positively influences many metabolic steps in a way that counteracts the potentially negative effects of a hypercaloric and hyperlipidic diet, which often characterizes the nutritional habits of western populations. In view of the results reported in this pre-clinical study, further clinical studies are warranted to confirm the effects of KO on human metabolism.

## Materials and Methods

### Materials

Bio-Rad protein assay kit and hydroxyapatite (Bio-Gel HTP) were purchased from Bio-Rad; Amberlite XAD-2, Dowex AG1-X8, Pipes, Triton X-100, Triton X-114, Sephadex G-75, 1,2,3-benzenetricarboxylate (1,2,3-BTA), cardiolipin, acetyl-CoA, phosphoenolpyruvate, ATP, NADH, NADPH, pyruvate kinase, lactate dehydrogenase, malonyl-CoA, 5,5′-dithio-bis (2-nitrobenzoic acid) (DTNB), carnitine, palmitoyl-CoA, cytochrome *c* and decylubiquinol (DBH_2_) were from Sigma; [1,5-^14^C] citrate was from Healthcare and egg yolk phospholipids were from Fluka; Krill oil (KO) was a generous gift of Aker BioMarine ASA (Oslo, Norway). Kits for the assay of triglycerides, total cholesterol and phospholipids were purchased from Futura System; kits for the determination of plasma insulin concentration and protein oxidation were purchased from Millipore; kit for assay of lipid oxidation was from Merck, whereas that for the assay of carnitine levels was purchased from Biovision. All other reagents were of analytical grade.

### Ethics Statement

This study was carried out in strict accordance with the European Committee Council 106 Directive (86/609/EEC) and with the Italian animal welfare legislation (art 4 and 5 of D.L. 116/92). The Italian Ministry of Health specifically approved this study.

### Animals

Male Sprague-Dawley rats (70–100 g) were obtained from Harlan (Carezzana, Italy) and housed individually in animal cages at a temperature of 22±1°C with a 12:12 hour light-dark cycle and 30–40% humidity. After 1 week of acclimatization, 10 rats were sacrificed (week 0) and 120 rats were divided into three groups of 40 animals each. The first group (control group) received a standard diet containing 6% fat (Global Diet 2018S from Harlan Teklad). The second group (HF group) received a diet with 35% fat (Diet TD.03584 from Harlan Teklad). The third group of animals (HF+KO group) was fed with the above reported HF diet supplemented with 2.5% KO. Diet composition is shown in Table 1. The animals were treated for 12 weeks and had *ad libitum* access to diets and water. Body weight, liver weight and food intake were recorded throughout the study. 10 animals in each group were sacrificed at week 4, 6, 8 and 12.

### Citrate transport in rat liver mitochondria

Rat liver mitochondria were prepared by following standard procedures and mitochondrial protein concentration was determined by the Bradford method [40]. Freshly isolated rat liver mitochondria were then loaded with malate. To this purpose, they were incubated (about 40–50 mg protein) at 20°C in 10 ml of 100 mM KCI, 20 mM Hepes, 1 mM EGTA, pH 7.0, in the presence of 2 mg/ml rotenone and 0.75 mM L-malate. After 2 min, 20–30 µmol/g protein of mersalyl was added in order to inhibit the dicarboxylate carrier. After 1 min at 20°C, the mitochondria were diluted with the above reported ice-cold buffer and centrifuged at 20,000 *g* for 5 min at 2°C. Then the reisolated mitochondria were washed once and finally resuspended in 1.5 ml of the same buffer.

[^14^C]citrate/malate exchange was determined by using the inhibitor stop method. This assay, carried out at 9°C, was initiated by the addition of 0.5 mM [^14^C]citrate and stopped by 12.5 mM 1,2,3-BTA. The mitochondria were immediately reisolated by centrifugation, washed once and acidified with 20% HClO_4_. The obtained supernatant was counted by liquid scintillation.

### Reconstitution of the citrate transport into liposomes

Rat liver mitochondria (10–15 mg proteins) were solubilized with a buffer containing 3% Triton X-100 (w/v), 20 mM Na_2_SO_4_, 1 mM EDTA, 10 mM Pipes, pH 7.0, at a final concentration of about 10 mg protein/ml. After incubation for 10 min at 2°C, the mixture was centrifuged at 25,000 *g* for 20 min at 2°C thereby obtaining a supernatant, referred to as mitochondrial extract. 600 µl of this extract (corresponding to about 6–7 mg protein), supplemented with 2 mg/ml cardiolipin, were applied to a cold hydroxyapatite column and eluted with a buffer containing 0.5% Triton X-100 and 5 mM citrate/NaOH, pH 7.0. The initial mixture used for the reconstitution experiments contained: 50 µl of hydroxyapatite eluate, 90 µl of 10% Triton X-114, 20 µl of 20 mg/ml cardiolipin, 100 µl of 10% phospholipids in the form of sonicated liposomes, 70 µl of 100 mM Pipes (pH 7.0) and 35 µl of 200 mM citrate in a final volume of 700 µl. After vortexing, this mixture was passed 15-times through the same Amberlite XAD-2 column, in order to obtain the proteoliposomes. Citrate present outside the proteoliposomes was removed by passing them through a Sephadex G-75 column preequilibrated with 50 mM NaCI and 10 mM Pipes (pH 7.0). The first 600 µl of the turbid eluate were collected, distributed in reaction vessels (180 µl) and used for the transport studies.

The assay of citrate transport was intiated by the addition of 0.5 mM [^14^C]citrate (unless otherwise indicated) to reconstituted proteoliposomes incubated at 25°C, and stopped after the indicated time by adding 20 mM 1,2,3-BTA. The radioactivity external to proteoliposomes was removed from each sample by chromatography on Dowex AG1-X8 columns and the internal radioactivity was measured by scintillation counting.

### Western blot analysis

The expression of the mitochondrial CIC was determined by western-blotting analysis. Polyacrylamide gel electrophoresis was performed in the presence of 0.1% SDS (SDS-PAGE) according to standard procedures. The mitochondrial proteins that have been separated by SDS-PAGE were transferred to a nitrocellulose membrane. For protein detection, antisera directed against the C-terminus of the rat liver CIC and against the mammalian porin were used at a dilution of 1∶3×10^3^. The immunoreacted proteins were detected by the peroxidase reaction, using *N*, *N′* Diamino Benzydine (DAB) and hydrogen peroxide.

### Assay of enzymes involved in fatty acid synthesis and oxidation

Rat liver cytosol was obtained by centrifuging the post-mitochondrial supernatant at 20,000 *g* for 20 min at 2°C. The pellet was discarded and the supernatant was then centrifuged at 105,000 *g* for 1 h. ACC activity was measured using a NADH-linked assay as described by [41]. FAS activity was measured by disappearance of absorbance of NADPH at 340 nm by adding 1 mg of cytosolic proteins to a mixture containing 85 mM acetyl-CoA, 0.126 mM NADPH, 100 mM phosphate, pH 6.5. The reaction was started with 0.115 mM malonyl-CoA [42].

Total carnitine palmitoyl-CoA transferase (CPT) activity was determined spectrophotometrically at 412 nm in freshly isolated rat liver mitochondria, essentially as described previously [43]. CPT I activity was calculated by subtracting the CPT activity that was insensitive to 100 mM malonyl-CoA from the total CPT activity experimentally determined. Liver carnitine levels were also determined using a commercial kit (L-carnitine assay kit, Biovision), according to the manufacturer's instructions.

### Mitochondrial respiration efficiency

Mitochondrial respiration (0.3 mg of mitochondrial protein/ml) was measured in a medium consisting of 220 mM sucrose, 20 mM KCl, 2.5 mM KH_2_PO_4_, 1 mM EDTA, 20 mM Hepes, 5 mM MgCl_2_, 2 µg/mL rotenone, 0.1% BSA and 5 mM K-succinate, pH 7.4, by a Clark oxygen electrode at 25°C. After 2 min, state 3 respiration was induced by the addition of 0.3 mM ADP. Respiratory control ratio (RCR) was calculated as the ratios of the rate of oxygen uptake in the presence of added ADP (state 3) to the rate observed when added ADP had been completely phosphorylated to ATP (state 4).

### Assay of mitochondrial complex III activity

Complex III activity was determined by measuring the reduction of oxidized cytochrome *c* at 550 nm. Rat liver mitochondria (40 µg protein) were incubated for 1 min at 30°C in a reaction medium containing 50 mM potassium phosphate (pH 7.2), 0.01% (wt/vol) Tween-20, 50 µM EDTA, 4 mM KCN and 40 µM oxidized cytochrome *c*. The reaction was initiated by adding the ubiquinol analog, decylubiquinol (DBH_2_), to a final concentration of 50 µM, and the rate of cytochrome *c* reduction was calculated from the absorbance increase at 550 nm. Specific activity was calculated as micromoles of cytochrome *c* reduced min^−1^ mg protein^−1^ with an extinction coefficient of 19.6 mM^−1^ cm^−1^.

### Lipid measurements in plasma and liver

For the determination of plasma lipids rats were starved overnight before sacrifice. Blood was collected and centrifuged to separate plasma (3,000 *g*, 10 min).

Liver lipids were extracted using a 1∶1 mixture of chloroform and methanol. The extracts were dried under nitrogen flow and resuspended in a suitable volume of 0.1% Triton X-100 before carrying out the individual lipid assays. Liver and plasma triglycerides, as well as cholesterol and phospholipid levels, were measured using commercial kits (Futura srl).

For histologic examination of the livers, intracardiac perfusion with 4% formaldehyde, freshly prepared from paraformaldehyde, was performed. Livers were then removed and post-fixed in formaldehyde for 1 h followed by three 1 h washing steps in phosphate-buffered saline (PBS). Liver pieces from the right ventral lobe were introduced into histological cassettes before paraffin wax embedding, cutted, mounted on slides and stained according to standard cresyl violet protocols.

### Phospholipid, cholesterol and fatty acid analysis of mitochondrial membranes

Total lipids were extracted from mitochondria (10 mg protein) by the Bligh and Dyer procedure [44]. The extracts were dried under a flow of N_2_ and resuspended in a proper volume of chloroform. Phospholipids were quantitatively assayed by determining inorganic phosphate by the procedure reported in Nakamura [45]. Cholesterol was extracted from mitochondria and assayed by HPLC [46].

To analyze fatty acids, liver mitochondria were saponified with ethanolic KOH for 2 h at 90°C. Fatty acids were extracted as reported in [46], and their corresponding methyl esters were prepared by *trans*-esterification with methanolic boron trifluoride (17% BF_3_) at 65°C for 30 min. Methyl esters of the fatty acids (FAMEs) were then analyzed by gas-liquid chromatography. The helium carrier gas was used at a flow rate of 1 ml min^−1^. FAMEs were separated on a 30 m×0.32 m HP5 (Hewlett Packard) capillare column. The injector and detector temperatures were maintained at 250°C. The column was operated isothermally at 150°C for 4 min and then programmed to 250°C at 4°C/min. Peak identification was performed by using known standards, and relative quantification was automatically carried out by peak integration.

### Glucose and insulin measurements

For the determination of blood glucose and plasma insulin, rats were starved overnight before sacrifice. Blood glucose concentration was determined using reactive strips and a commercial glucometer (One Touch Basic Plus, LIFESCAN, Johnson & Johnson). Plasma insulin concentration was analyzed with commercial enzyme-linked immunosorbent assay kits (Millipore EZRMI-13K).

### Determination of oxidative damage

Lipid peroxidation levels in liver samples were determined using a lipid hydroperoxide assay kit (Merck) which measures the redox reactions with ferrous ions.

The protein carbonyl contents in liver tissue lysates were detected by the OxyBlot Protein Oxidation Detection Kit (Millipore), according to the manufacturer's instructions. The carbonyl groups in the protein side chains were derivatized to 2,4-dinitrophenylhydrazone (DNPhydrazone) by reaction with 2,4-dinitrophenylhydrazine (DNPH). The DNP-derivatized protein samples were separated by polyacrylamide gel electrophoresis followed by western blotting. DNP protein bands were visualized by chemiluminescence. Oxyblot images were analyzed using an imaging densitometer.

### Statistical analysis

Experimental data represent the means ± SD. The data were analyzed by one-way ANOVA and a Tukey-Kramer *post hoc* analysis was used to detect significant differences between the means at a level of *P*<0.05.
